# Effects of preoperative anxiety level on pain level and joint functions after total knee arthroplasty

**DOI:** 10.1038/s41598-023-48291-2

**Published:** 2023-11-27

**Authors:** Onur Varış, Gökhan Peker

**Affiliations:** University of Healt Sciences, Trabzon Faculty of Medicine, Kanuni Training and Research Hospital, Trabzon, Turkey

**Keywords:** Diseases, Medical research

## Abstract

This study examined the effect of preoperative anxiety level on postoperative pain, opioid and nonopioid analgesic use requirements and joint function in patients undergoing primary knee arthroplasty for knee osteoarthritis. 106 patients were included in the study. Situational anxiety score (STAI-S) and trait anxiety score (STAI-T) were divided into two groups as below and above 40. Visual analog scale (VAS), Lysholm scores, and anxiety levels were assessed preoperatively, at the third and sixth month postoperatively to investigate their effects on the use of opioid and non-opioid drugs during the postoperative period. For those with low situational and trait anxiety scores, the median duration of hospital stays was significantly shorter compared to those with high anxiety scores. In groups where anxiety levels were initially low, the median anxiety levels at the postoperative 3rd month were also observed to be lower compared to the other group. In the same groups, the median VAS values and the median Lysholm knee scores were significantly better both preoperatively and at the postoperative 3rd month compared to the other group. There was no significant difference in Lysholm score between the two groups at the 6th postoperative month. Similarly, in the preoperative period, groups with low anxiety levels had a significantly lower median usage of both opioids and non-opioids compared to the other group. These findings suggest that high preoperative anxiety may have a negative effect on functional outcomes in the early postoperative period, but this effect disappeared at 6 months. It can be concluded that reducing preoperative anxiety may have a positive effect on early outcomes. We believe that preoperative psychological evaluation and treatment can increase the satisfaction level in patients undergoing total knee arthroplasty (TKA).

## Introduction

Osteoarthritis (OA) is a chronic degenerative disease characterized by biochemical and morphological changes in the synovial membrane and joint capsule, leading to subchondral sclerosis, osteophyte formation, and erosion in joint cartilage. Its prevalence increases with age, and it can significantly impair an individual's quality of life due to pain and disabilities^1^. Gonarthrosis is osteoarthritis of the knee joint and is the most common form of osteoarthritis^2^. In epidemiologic studies conducted in various parts of the world, it has been reported that 10–30% of people over the age of 65 have symptomatic knee OA^3^. In a prevalence study conducted in Türkiye, the prevalence of symptomatic knee OA in the population aged 50 years and older was 14.8% and was reported as 22.5% in women and 8% in men^4^.

Globally, the incidence of gonarthrosis has consistently increased over the last two decades and is 150–200/100,000 in industrialized countries^5,6^. Due to the increasing incidence of gonarthrosis, the global demand for total knee arthroplasty (TKA) is rising dramatically. It is expected that the number of primary TKA procedures will increase by 85% (to 1.26 million procedures) by 2030^7,8^. TKA is an effective treatment method to improve the functions and quality of life of patients with end-stage osteoarthritis in the knee^9,10^. The stress and anxiety from the surgery, as well as the pain and challenges in daily life activities, can affect post-operative patients' needs for analgesics and joint functions^11^. In a study conducted by Heuts et al., it was found that pain-related fear of movement was present in patients with knee OA and that this was related to joint functions^12^. The association between higher anxiety and worse joint function supports the importance of considering anxiety as well as physical impairments in the evaluation of patients with knee OA^13,14^.

In our study, we aimed to investigate the effects of preoperative anxiety levels of patients diagnosed with advanced primary gonarthrosis, who underwent TKA, on postoperative pain intensity, opioid and non-opioid analgesic needs, and joint functions. We believe that the TKA operations, which hold a significant place in daily orthopedic practice, may pose a significant financial issue considering both the patient's hospitalization duration and postoperative analgesic use, as well as potential physical therapy requirements. We anticipate that reducing preoperative anxiety levels will decrease the use of analgesics and increase patients' motivation, enhancing their adherence to exercise programs, leading to positive effects on joint functions.

## Material and method

Our study was approved by the ethics committee of our hospital (Approval was obtained from the Clinical Research Ethics Committee of the Kanuni Training and Research Hospital on 04.04.2021. Our study was conducted in accordance with the Declaration of Helsinki.) and those who were scheduled for TKA surgery and agreed to participate voluntarily were included.Informed consent was obtained from all patients included in the study.

106 patients were included in the study. Considering the change in the Situational Anxiety Score (STAI-T) variable over time, a post-hoc power analysis was performed, and the study was found to be very robust. [The Posthoc Power Analysis was performed with a Type I error rate (alpha) of 0.05, a sample size of 106, an effect size of 4.15, and considering a two-sided alternative hypothesis (H_1_). Using the Wilcoxon Signed-Rank test, the calculated power (1-beta) was determined to be 1 (100%)] Patients with known psychiatric diseases, those who underwent revision surgeries, those with complications (excluding chronic-long-term pain), those previously taking medications like pregabalin or gabapentin that could affect pain perception, and those who had postoperative pain pump applications or epidural blocks were excluded from the study. In addition, patients who previously had orthopedic surgical interventions on the lower extremity joints were excluded. Furthermore, morbidly obese patients with a body mass index (BMI > 40) were also excluded.

After recording the patients' demographic data, they were informed about the study and their voluntary consents were obtained. Subsequently, in the preoperative period, Visual Analog Scale (VAS), Lysholm score, Situational Anxiety Score (STAI-S), and STAI-T were filled out. The amount of postoperative analgesic and opioid use of patients was recorded. During the 3rd and 6th month post-operative check-ups, VAS, Lysholm, and both STAI-S and STAI-T were filled out again.

Patients were operated on under a pneumatic tourniquet. During the surgery, a standard midline incision with a medial parapatellar approach was used. The patients' comorbidities, operation durations, the anesthesia methods applied, and the opioids and non-opioid analgesics used in the postoperative period were recorded. All patients were implanted with the same brand of knee prosthesis that includes a mobile insert preserving the posterior cruciate ligament by a single experienced surgeon.

Patients were routinely monitored in the post-anesthetic care unit (PACU) on the first post-operative night and were moved to their beds the next day. Walking and movement exercises were started on the first postoperative day. The patients were independently mobilized and discharged home after their pain was controlled, 90° knee flexion was achieved and their wounds were confirmed to be free of any problems. Before discharge, our clinic's physiotherapist provided all patients with detailed explanations about the necessary exercises and gave them a home-based exercise program. Patients were called monthly to check their compliance with the exercise program. Patients experiencing problems with exercise program adherence were invited for control, and compliance issues were addressed with the assistance of a physiotherapist. All patients were called for routine monthly check-ups after discharge. During the third and sixth month check-ups, VAS, Lysholm, STAI-S, and STAI-T scores were repeated.

The same physical therapy program was applied to the patients during their hospitalization. Pain statuses were evaluated, and as a post-operative non-opioid analgesic, a single agent diclofenac sodium 75 mg/3 ml was applied intravenously, and as an opioid, tramadol hydrochloride 100 mg/2 ml was administered intravenously.

After gathering all patients' data, the patients were divided into two groups based on their pre-operative STAI-S scores. Patients with a preoperative STAI-S score of 40 or above (including 40) were classified as having a high anxiety level (Group s1). Others were included in the group with low anxiety levels (Group s2). Both groups' demographic data (education status, age, etc.), admission and surgery durations, Lysholm scores (pre-postoperative third and sixth months), VAS scores (pre-postoperative third and sixth months), postoperative analgesic uses (opioid, nonopioid), and changes in anxiety scores (pre-postoperative third and sixth months) were compared. The same comparison process was repeated based on STAI-T scores. Patients with a preoperative STAI-T score of 40 or above (including 40) were classified as having a high anxiety level (Group t1). Others were included in the group with low anxiety levels (Group t2).

### Statistical analysis

Statistical analysis of the data was performed using the IBM SPSS Statistics 25 software package. The normal distribution of the data was examined using the Shapiro–Wilk test. Descriptive statistics of the data were stated as mean ± standard deviation for normally distributed continuous variables, as [median (minimum: maximum)] for non-normally distributed variables, and as frequency and percentage [n(%)] for categorical variables. For determining the difference between independent group averages for normally distributed continuous variables, the Independent Samples t-Test was used. For non-normally distributed continuous variables, the Mann–Whitney U test was used. Comparisons of time-dependent measurements between groups for continuous variables were done based on the initial value (Baseline or pre-op) through the difference score (difference = (final value [post-op] – initial value [pre-op]). For within-group comparisons of time-dependent measurements of non-normally distributed continuous variables, the Wilcoxon Signed Rank Test was used. For analyzing categorical data, the Pearson chi-square and Fisher's exact chi-square tests were used. The significance level was set at α = 0.05. Statistically significant significance values are bolded in the tables.

## Results

The 106 patients included in the study were grouped according to their STAI-S and STAI-T scores.

The gender data of patients grouped by two different anxiety scores are given in Tables 1 and 2.

**Table 1:** Comparison of gender variables between groups according to STAI-S classification.

Variable	STAI-S high (n = 72) (group s1)	STAI-S low (n = 34) (group s2)	P
Age (mean ± std. deviation)	65.25 ± 6.52	66.03 ± 6.63	0.569^&^
Gender			
Female n (%) Male n (%)	70 (97.2%)	26 (76.5%)	0.002*
	2 (2.8%)	8 (23.5%)	

*Pearson chi-square or Fisher exact chi-square tests.

^&^Independent samples t test.

Significant value is in bold.

**Table 2:** Comparison of Gender Variables Between Groups According to STAI-T Classification.

Variable	STAI-S high (n = 49) (group t1)	STAI-T low (n = 56) (group t2)	P
Age (mean ± std. deviation)	65.80 ± 6.92	65.21 ± 6.28	0.653^&^
Gender			
Female n (%)	50 (100%)	46 (82.1%)	0.002*
Male n (%)	0 (0%)	10 (17.9%)	

*Pearson chi-square or Fisher exact chi-square tests.

^&^Independent samples t test.

Significant value is in bold.

The number of male individuals in Group s1 and Group t1 was found to be significantly lower (Tables 1 and 2).

The comparison of the duration of surgery, length of hospitalization, VAS values, Lysholm scores, and the amount of opioid and nonopioid use according to the grouping made according to the STAI-S score is given in Table 3.

**Table 3:** Intergroup comparison of variables according to STAI-S classification.

Variable	STAI-S high (n = 72) (Group s1)	STAI-S low (n = 34) (Group s2)	P
Anesthesia type			
Spinal n (%)	65 (90.3%)	33 (97.1%)	0.432*
General n (%)	7 (9.7%)	1 (2.9%)	
Operation time (min) [median (min:max)]	65 (45:80)	55 (50:75)	**0.002**^**#**^
Length of hospitalization (days) [median (min:max)]	5 (3:7)	4 (2:6)	** < 0.001**^**#**^
Preoperative VAS [median (min:max)]	8 (6:9)	8 (6:9)	**0.004**^**#**^
Postoperative 3rd month VAS [median (min:max)]	5 (3:7)	4 (3:5)	**0.003**^**#**^
Postoperative 6th month VAS [median (min:max)]	4 (2:5)	3 (2:5)	0.115^**#**^
Preoperative Lysholm [median (min:max)]	48 (28:68)	58 (34:68)	**0.017**^**#**^
Postoperative 3rd month Lysholm [median (min:max)]	67.5 (48:84)	74.5 (51:86)	**0.015**^**#**^
Postoperative 6th month Lysholm [median (min:max)]	72 (54:88)	75.5 (54:87)	0.059^**#**^
Preoperative STAI-S [median (min:max)]	45.5 (41:54)	37 (31:39)	** < 0.001**^**#**^
Postoperative 3rd month STAI-S [median (min:max)]	40 (31:49)	34 (30:43)	** < 0.001**^**#**^
Postoperative 6th month STAI-S [median (min:max)]	36 (28:46)	33 (26:38)	** < 0.001**^**#**^
Preoperative 3rd month STAI-S difference* [median (min:max)]	−6 (−18:2)	−2 (−7:4)	** < 0.001**^**#**^
Preoperative 6th month STAI-S difference*[median (min:max)]	−9 (−9:2)	−4 (−11:2)	** < 0.001**^**#**^
Preoperative STAI-T [median (min:max)]	42 (34:52)	37 (32:43)	** < 0.001**^**#**^
Postoperative 3rd month STAI-T [median (min:max)]	40 (32:48)	37 (30:45)	** < 0.001**^**#**^
Postoperative 6th month STAI-T [median (min:max)]	39 (30:47)	36.5 (33:39)	** < 0.001**^**#**^
Preoperative 3rd month STAI-T difference* [median (min:max)]	−1 (−11:6)	0 (−9:11)	**0.011**^**#**^
Preoperative 6th month STAI-T difference* [median (min:max)]	−3 (−14:6)	0 (−9:6)	**0.025**^**#**^
Nonopioid [median (min:max)]	20 (14:34)	13.5 (6:24)	** < 0.001**^**#**^
Opioid [median (min:max)]	7 (2:12)	3 (0:6)	** < 0.001**^**#**^

*Pearson Chi-square or Fisher exact Chi-square tests.

#Mann Whitney U test.

Significant values are in bold.

In Group s2, the median surgery time and hospitalization durations were significantly lower compared to Group s1. Similarly, in Group s2, the median VAS and lysholm scores, both preop and at the 3rd month postop, were significantly better than the other group. By the 6th month postoperatively, there was no significant difference between the two groups in terms of median VAS and Lysholm scores. Group s2's median STAI-S scores during postoperative follow-ups were found to be significantly better than the other group. Throughout the follow-ups, Group s2's median STAI-T scores were also significantly better compared to the other group. In a similar vein, both opioid and non-opioid median usage values in Group s2 were significantly lower compared to the other group (Table 3).

The comparison of the duration of surgery, length of hospitalization, VAS values, Lysholm scores, and the amount of opioid and nonopioid use according to the grouping made according to the STAI-T score is given in Table 4.

**Table 4:** Intergroup comparison of variables according to STAI-T classification.

Variable	STAI-T high (n = 49) (group t1)	STAI-T low (n = 56) (group t2)	P
Anesthesia type			
Spinal n (%)	46 (92%)	52 (92.9%)	1.000*
General n (%)	4 (8%)	4 (7.1%)	
Operation time [median (min:max)]	65 (45:80)	55 (45:75)	0.065^**#**^
Length of hospitalization [median (min:max)]	5 (3:7)	4 (2:6)	** < 0.001**^**#**^
Preoperative VAS [median (min:max)]	8 (7:9)	8 (6:9)	** < 0.001**^**#**^
Postoperative 3rd month VAS [median (min:max)]	5 (3:7)	4 (3:6)	**0.004**^**#**^
Postoperative 6th month VAS [median (min:max)]	4 (2:5)	3 (2:5)	**0.030**^**#**^
Preoperative Lysholm [median (min:max)]	48 (28:68)	54 (28:68)	0.368^**#**^
Postoperative 3rd month Lysholm [median (min:max)]	66 (48:84)	72 (50:86)	**0.039**^**#**^
Postoperative 6th month Lysholm [median (min:max)]	72 (54:86)	74 (54:88)	0.088^**#**^
Preoperative STAI-S [median (min:max)]	46 (38:54)	39 (31:51)	** < 0.001**^**#**^
Postoperative 3rd month STAI-S [median (min:max)]	41 (32:49)	34.5 (30:45)	** < 0.001**^**#**^
Postoperative 6th month STAI-S [median (min:max)]	37 (28:46)	34 (26:42)	** < 0.001**^**#**^
Preoperative 3rd month STAI-S difference* [median (min:max)]	−6 (−14:2)	−4 (−18:4)	0.141^**#**^
Preoperative 6th month STAI-S difference*[median (min:max)]	−9 (−19:2)	−5 (−16:2)	**0.013**^**#**^
Preoperative STAI-T [median (min:max)]	43 (40:52)	37 (32:39)	** < 0.001**^**#**^
Postoperative 3rd month STAI-T [median (min:max)]	41 (35:48)	37 (30:45)	** < 0.001**^**#**^
Postoperative 6th month STAI-T [median (min:max)]	40 (31:47)	37 (30:45)	**0.002**^**#**^
Preoperative 3rd month STAI-T difference* [median (min:max)]	−2 (−11:5)	0 (−9:11)	** < 0.001**^**#**^
Preoperative 6th month STAI-T difference* [median (min:max)]	−5 (−14:4)	1 (−9:6)	** < 0.001**^**#**^
Nonopioid [median (min:max)]	23 (14:34)	16 (6:23)	** < 0.001**^**#**^
Opioid [median (min:max)]	7 (2:12)	3 (0:8)	** < 0.001**^**#**^

*Pearson chi-square or Fisher exact chi-square tests.

#Mann Whitney U test.

Significant values are in bold.

In Group t2, the median hospitalization duration was significantly shorter than in Group t1. The median VAS values of group t2 were better than the other group in all follow-ups. While there was no difference in the median preop Lysholm scores between the groups, Group t2's median Lysholm score was better than the other group at the 3rd month postop, but this difference disappeared by the 6th month postop. In Group t2's postoperative follow-ups, the median STAI-T score was significantly better compared to the other group. Similarly, both opioid and non-opioid median usage values in Group t2 were significantly lower compared to the other group (Table 4).

The change in VAS, Lysholm, STAI-S and STAI-T scores of the whole study group over time is given in Table 5.

**Table 5:** Comparison of in-group time dependent measurements of variables.

Variable	Preoperative (n = 106)	Postoperative 3rd month (n = 106)	Postoperative 6th month (n = 106)	P**
VAS [median (min–max)]	8 (6**–**9)	5 (3–7)	4 (2–5)	(Pre-post 3rd month) < 0.001 (Pre-post 6th month) < 0.001
Lysholm [median (min–max)]	51 (28 **–**68)	68 (48 –86)	73 (54–88)	(Pre-post 3rd month) < 0.001 (Pre-post 6th month) < 0.001
STAI-S [median (min–max)]	44 (31**–** 54)	37 (30–49)	35 (26–46)	(Pre-post 3rd month) < 0.001 (Pre-post 6th month) < 0.001
STAI-T [median (min–max)]	39 (32**–**52)	39 (30–48)	38 (30– 47)	(Pre-post 3rd month) < 0.014 (Pre-post 6th month) < 0.001

**Wilcoxon signed rank test.

For all patients included in the study, median preoperative values for VAS, STAI-S, and STAI-T scores significantly decreased during postoperative follow-ups, while the median preoperative values for Lysholm scores showed significant improvement (Table 5).

## Discussion

In our study, we observed that in groups with higher anxiety scores (Group s1 and Group t1), the use of opioids and non-opioids was higher, and their Lysholm scores were worse in their 3-month follow-ups. Additionally, these patients had longer hospital stays. Even though the median surgery time in Group s1 was statistically significantly higher based on STAI-S grouping, we do not believe there's a clinically relevant explanation.

Two prospective studies that used validated mental health surveys to evaluate preoperative depression prevalence in TKA patients reported prevalence rates of 22.5% and 26%^15,16^. The relationship between the outcome of TKA surgery and psychological disorders has always been of interest. In their first report of a study of 116 patients who underwent primary total knee replacement between 1998 and 2000, Brander et al. found that those with preoperative anxiety and depression were more likely to suffer from pain at one-year follow-up^17^. In this regard, depression and anxiety constitute a significant concern in patients undergoing TKA. Our findings also align with these studies, indicating that patients with high anxiety scores had increased analgesic use. Our results are consistent with previous studies showing that higher preoperative depression and anxiety levels predict worse clinical outcomes and higher pain levels after TKA^18–21^.

Of the patients included in the study, 96 were female and 10 were male. Gonarthrosis is more common in females. The number of male participants in our study was considerably lower compared to females. The median STAI-S and STAI-T scores of males in our study were statistically significantly better than females. In fact, anxiety and stress disorders are more common in women^22^. Moerman et al. evaluated the relationship between gender and anxiety in a study of 320 patients and reported that anxiety was significantly higher in women^23^. Our study coincides with the literature in this respect. There was no significant difference between the groups in our other demographic data.

In Group s2, both median surgery durations and median hospitalization times were significantly shorter compared to Group s1. In our grouping according to STAI-T, there was no statistically significant difference between the operation times (Group t1 and Group t2). We think that an increase in the duration of surgery can increase the postoperative analgesic need and impair functional outcomes. Indeed, an extended surgical duration is a risk factor for surgical site infection, and surgical site infections can lead to adverse outcomes in TKA^24–27^. XinPan et al. found that patients with psychiatric comorbidity had shorter hospitalization periods after TKA^28^. Although we excluded patients diagnosed with a psychiatric disorder, our hospitalization durations in the group with high anxiety scores are contrary to the results of XinPan et al. We found that the length of hospitalization in these patients was significantly higher as in the study by Ali et al.^29^. This may be explained by the fact that patients with high levels of anxiety may feel safer in the hospital and may be more afraid of going home early. Likewise, the need for analgesics was higher in this group of patients compared to the other group, so the postoperative pain of these patients could be controlled later and they were discharged later.

In both Group s2 and Group t2, median VAS scores were better both preoperatively and at the 3rd-month postoperative follow-ups. The results in our study support the study of Ali et al. on dissatisfaction (considering pain scores) after TKA^29^.

In Group s2, the median Lysholm knee scores were significantly better both preoperatively and at the 3rd-month postop compared to the other group. There was no significant difference in Lysholm score between the two groups at the 6th postoperative month. Even though we have not conducted a correlation analysis on this, we believe that with the reduction of pain and anxiety in the postoperative period, knee functions may improve. Many studies^17,18,30–33^ have found that a higher baseline level of anxiety and depression predicts a worse outcome. Brander et al. found that increased preoperative pain levels and preoperative depression were predictors of worse AKSS (American Knee Society Score) scores, but this difference disappeared in a 5-year follow-up^34^. The reason for this might be that patients avoid exercising due to pain; moreover, patients showing symptoms of depression might have difficulty adhering to exercise programs.Contrarily, Riddle and colleagues could not find a relationship between psychological variables (anxiety and depression) and low WOMAC (Western Ontario and McMaster Universities Osteoarthritis Index) outcome scores^35^. However, the focus of Riddle and colleagues' study was on a subgroup of patients who had poor outcomes after knee arthroplasty. Their follow-up durations were relatively short. The results they obtained might be related to this^34^. In our study, high anxiety levels seem to negatively affect knee functions in the first three months. In this regard, our study supports the findings of Brander and his colleagues^35^.

Looking at the literature, previous studies have shown that most patients with good functional outcomes after TKA experienced a significant relief in symptoms of depression and anxiety^30^. In our study, groups with low anxiety scores (Group s2 and Group t2) had significantly better median Lysholm scores compared to other groups in the first 3 months. In relation to this, several studies have shown that comorbid depression or anxiety affects not only short-term outcomes after TKA, but also long-term outcomes and may be an early precursor of pain in the postoperative period^36,37^. However, despite our study having a short follow-up duration, by the 6th-month follow-up, the median Lysholm scores of Group s2 and Group t2 were not significantly different from the other groups.

Even though the primary aim of our study was not to compare the efficacy of anxiety scores, and we have not conducted a correlation analysis on this, we came to the conclusion that the two anxiety scores predicted similar outcomes.

Some researchers believe that depression is associated with a chronic, low-grade inflammatory response and activation of the compensatory anti-inflammatory reflex system^38^. One study found that depression was associated with higher nonsteroidal anti-inflammatory drug use and that anxious patients showed higher opioid consumption after primary TKA^39^. Other studies have also reached similar conclusions with patients who have major depressive disorders, reporting increased pain perception and opioid consumption following TKA^40^. Namba et al. found that anxiety, depression, back pain and non-specific chronic pain may prolong postoperative opioid use after TKA^41^. In our study, groups with low anxiety scores had both opioid and non-opioid usage significantly lower compared to the other group. Our study aligns with other studies in the literature in this respect.

We also observed that VAS, STAI-S, STAI-T and Lysholm scores improved significantly in the ongoing follow-up of all patients included in the study.

One of the limitation of our study is that we did not have data to examine other behavioral factors, such as self-efficacy and coping skills, so we could not include them in the evaluation. Additionally, we did not record data on the patients' cognitive statuses. Another limitation is the short duration of our follow-up. This is due to difficulties patients faced in accessing healthcare services caused by the curfews imposed during the COVID-19 pandemic.

We believe that studies excluding patients who were not previously diagnosed but were consulted with a psychiatric expert in the preoperative period could be more guiding on this matter.

## Conclusion

Although we cannot claim causality in our results, the amount of analgesic use was less in patients with low anxiety levels and their joint functions were beter than the other group.We believe that preoperative psychological evaluation and treatment will reduce analgesic use in TKA patients, positively affect knee functions, and consequently reduce the cost of treatment per patient.

## Data Availability

The datasets used and/or analysed during the current study available from the corresponding author on reasonable request.
